# Evolutionary Mechanism of Immunological Cross-Reactivity Between Different GII.17 Variants

**DOI:** 10.3389/fmicb.2021.653719

**Published:** 2021-04-06

**Authors:** Yueting Zuo, Liang Xue, Junshan Gao, Yingyin Liao, Yanhui Liang, Yueting Jiang, Weicheng Cai, Zhiwei Qin, Jiale Yang, Jumei Zhang, Juan Wang, Moutong Chen, Yu Ding, Qingping Wu

**Affiliations:** ^1^School of Bioscience and Bioengineering, South China University of Technology, Guangzhou, China; ^2^Guangdong Provincial Key Laboratory of Microbial Safety and Health, State Key Laboratory of Applied Microbiology Southern China, Institute of Microbiology, Guangdong Academy of Sciences, Guangzhou, China; ^3^Department of Laboratory Medicine, First Affiliated Hospital of Guangzhou Medical University, Guangzhou, China

**Keywords:** norovirus, GII.17, capsid protein, immunogenicity, evolutionary mechanism

## Abstract

Human norovirus is regarded as the leading cause of epidemic acute gastroenteritis with GII.4 being the predominant genotype during the past decades. In the winter of 2014/2015, the GII.17 Kawasaki 2014 emerged as the predominant genotype, surpassing GII.4 in several East Asian countries. Hence, the influence of host immunity response on the continuous evolution of different GII.17 variants needs to be studied in depth. Here, we relate the inferences of evolutionary mechanisms of different GII.17 variants with the investigation of cross-reactivity and cross-protection of their respective antisera using the expression of norovirus P particles in *Escherichia coli*. The cross-reactivity assay showed that the antisera of previous strains (GII.17 A and GII.17 B) reacted with recent variants (GII.17 C and GII.17 D) at high OD values from 0.8 to 1.16, while recent variant antisera cross-reacting with previous strains were weak with OD values between 0.26 and 0.56. The cross-protection assay indicated that the antisera of previous strains had no inhibitory effect on recent variants. Finally, mutations at amino acids 353–363, 373–384, 394–404, and 444–454 had the greatest impact on cross-reactivity. These data indicate that the recent pandemic variants GII.17 C and GII.17 D avoided the herd immunity effect of previous GII.17 A and GII.17 B strains through antigenic variation.

## Introduction

Norovirus (NoV) is one of the most leading causes of epidemic acute gastroenteritis (AGE) worldwide, affecting people of all ages and asymptomatic transmission is common (Quee et al., 2020). It is estimated that there are approximately 699 million norovirus illnesses and 219,000 deaths each year, resulting in $4.2 billion in health system costs and $60.3 billion in societal costs (Bartsch et al., 2016). In recent years, the NoV infection rate of sporadic diarrhea in China has ranged from 8.2 to 28.57% (Xue et al., 2019). The main routes of NoV transmission are foodborne, waterborne, airborne, and contaminated surfaces or infected persons (Alsved et al., 2020). The vomitus and feces of infected subjects contain a large number of virions, whereas as low as 10 infectious particles are required to cause AGE (Teunis et al., 2010). It can be seen that NoV is a threat to public health.

NoV in the family Caliciviridae is nonenveloped single-stranded positive-sense RNA viruses. The genome is 7.5–7.7 kb in length, which contains three open reading frames (ORF1, ORF2, and ORF3). ORF2 encodes the major structural protein VP1 and ORF3 encodes the minor structural protein VP2. X-ray crystallographic structure studies show that the capsid protein VP1 has a shell (S) and a protruding (P) domain (Prasad et al., 1999). The S domain surrounds the viral RNA, while the P domain is linked to the S domain *via* a flexible hinge (Vinjé, 2015). P domain is further divided into the central P1 subdomain and a highly variable P2 subdomain, with the latter, which is essential for virion binding with potential neutralizing antibodies and histo-blood group antigens (HBGAs; Tan et al., 2003). Structural changes of the capsid may lead to the possible emergence of a new phenotype (Mikael et al., 2003).

In 2014/2015, the sudden outbreaks of GII.17 Kawasaki variant have raised widespread concern (Xue et al., 2016b; da Silva Ribeiro de Andrade et al., 2018). GII.17 genotype was grouped into four different clusters (clusters A–D) based on the maximum likelihood analysis (Xue et al., 2016c). Strains in clusters A ranged from 1978 to 2015 and in cluster B emerged from 2005 to 2009. Strains detected after 2012 were divided into cluster C and cluster D (Sang and Yang, 2018). Distances of strains in cluster A ranged from 2.1 to 2.6% indicated the stability of the *VP1* gene over a long period of time. However, the distances between cluster B and cluster A were 7.0–8.0%. The distances between recent strains (cluster C and D) to earlier strains (cluster A and B) increased by 10.0–15.0%, indicating that the recently emerged GII.17 strains were new variants (Chen et al., 2015). GII.17 genotype evolved at a rate of 1.68×10^−3^ (95% HPD: 0.79×10^−3^ – 2.65×10^−3^) nucleotide substitutions/site/year (Sang and Yang, 2018).

The lack of cell culture systems or small animal models for human diseases severely hinders the development of vaccines and therapeutic agents. Moreover, these viruses are highly heterogeneous and undergoing antigenic variation in response to human immunity, further complicating our understanding of the complex immune interactions that regulate susceptibility and disease (Debbink et al., 2012b). Recent studies have shown that HBGAs are the attachment factors of human norovirus, which are critical in determining NoV host susceptibility and epidemiology (Van Trang et al., 2014; Nordgren and Svensson, 2019). In addition, the blockade assay has been used as a surrogate neutralization assay, and pig gastric mucin (PGM) type III is used as a substrate (Harrington et al., 2002; Lindesmith et al., 2011, 2012). NoV specific antibodies that block the binding of P particles to HBGAs are considered as the best correlate of protection against NoV.

**Figure 1:**
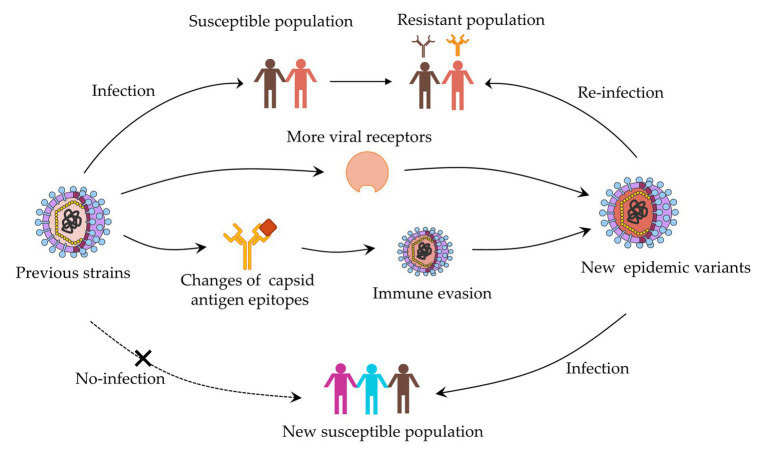
Evolution mechanism of norovirus based on capsid protein variation.

On the capsid surface, amino acids surrounding the binding pocket evolve rapidly, altering HBGA and antibody binding affinity (Debbink et al., 2012a). On one hand, these viruses may evolve to bind with different HBGAs, which indicate that individuals resistant to one pandemic strain may be susceptible to the other. On the other hand, minor sequence and structural differences allow these viruses to escape pre-existing herd immunity, ultimately leading to the emergence of new strains, which are competent to infect the same individuals who had been infected before (Donaldson et al., 2008; Figure 1). While the epidemiology and genetic analyses of new GII.17 variants were reported (Xue et al., 2016a,c), and further study to probe the emergence and its high prevalence is significant. Extensive antigenic relationships among NoV GII.17 variants are still unknown and the complex relationship between host protective immunity and virus antigenic heterogeneity are the primary obstacles to vaccine development. In this study, we systematically investigated the antigenic and ligand-binding characteristics of the four time-ordered GII.17 strains and found potentially critical amino acids that contribute to antigenic variation.

## Materials and Methods

### Ethical Statement

All the animal protocols performed in this study were approved by the Guangdong Institute of Microbiology Laboratory Animal Ethics Committee (permission number GT-IACUC202004271).

### Expression and Purification of P Particles in *Escherichia coli*

Four clusters of representative capsid P proteins of NoV GII.17 strains were prepared by *E. coli* prokaryotic expression system (Tan and Jiang, 2005). A cysteine-containing peptide was linked to the N or C terminus of the P domains to promote P-particle formation by forming intermolecular disulfide bridges (Tan and Jiang, 2005). Briefly, the sequences encoding the P domains of NoV GII.17 A-D were amplified by PCR and cloned into the expression vector pGEX-4T-1. After sequence confirmation by sequencing, the bacterial cultures were expressed in *E. coli* BL21 (DE3) with an induction of 0.5 mM isopropyl-β-D-thiogalactopyranoside (IPTG) at 20°C for 20 h. Purification of P protein-glutathione S-transferase (GST) fusion proteins was performed using GSTrap FF. The GST tag was removed by thrombin cleavage for 16 h at 22°C. SDS-PAGE verified the purity of P-protein. Finally, we used a BCA assay kit to measure the concentration of P particle.

### Production of Capsid P Protein of GII.17 D Mutants

Capsid P protein of NoV GII.17 D mutants were produced by our laboratory (unpublished data). In brief, after multiple alignments of GII.17 NoVs capsid protein sequences, six mutations were designed on hyper-variable regions by changing these amino acids to alanine, which located in 293-300aa, 342-349aa, 353-363aa, 373-384aa, 394-404aa, and 444-454aa. All mutants were expressed and purified as described above.

### Mice Immunization and Sample Collection

Female BALB/c mice purchased from the Laboratory Animal Center of Southern Medical University were divided into four groups of three mice. At 7 weeks old, the mice were immunized four times by subcutaneous injection of 30 μg of GST-P domain fusion protein in Freund’s adjuvant at weeks 0, 2, 4, and 5. PBS of the equivalent volume was injected as a control. P particles were emulsified in Freund’s complete adjuvant in the first injection and Freund’s incomplete adjuvant in the other three injections. Blood samples were collected at weeks 0 (pre-immune serum), 2, 4, 5, and 6. Centrifuging the microtainer tubes in a microcentrifuge to separate serum from the whole blood. Serum samples were transferred to a 1.5-ml microcentrifuge tube and stored at −20°C for later use.

### Enzyme Linked Immunosorbent Assay

Antigen-specific and cross-reactive antibody responses were determined by indirect enzyme-linked immunosorbent assay (ELISA). Ninety-six-well microtiter plates were coated with P particle 2 μg/ml at 4°C overnight and blocked with 5% skimmed milk at 37°C for 2 h. Serum samples were added as 2-fold dilution series starting from 1:200 dilution, followed by incubation for 1 h at 37°C. Subsequently, the plates were incubated with the secondary HRP-conjugated goat anti-mouse IgG antibody (diluted 1:3,000) for 30 min at 37°C. Tetramethylbenzidine (TMB) was added as the peroxidase chromogenic substrate to react for 7 min at 37°C. After stopping the substrate reaction with 2M H_2_SO_4_ and the optical density (OD) values were measured at 450 nm. A sample was considered as positive if the net OD value was above the set cut-off and at least 0.2 OD (Uusi-Kerttula et al., 2014), and the highest reciprocal serum dilution that yielded absorbance >2-fold over the background value was taken as the end-point titer. Geometric mean titers (GMTs) with 95% confidence intervals (CIs) for each immunization group were counted from individual mice end-point titers. Sera dilution with OD450 value of 0.8–1.2 was used for sera antibody cross-reactivity.

### HBGAs Binding Assay

The HBGAs binding assay was carried out with the P particle and two different sources of HBGAs: human saliva (type A, B, O, and non-secretor) and PGM type III. Microtiter plates were coated with saliva samples (1:1,000 dilution) and PGM at 4°C overnight. After blocking with 5% skimmed milk, the coated saliva samples and PGM were incubated with P particle at concentrations of 1 μg/ml for 1 h at 37°C. Subsequently, the plates were incubated with antisera from mice immunized with P particles for 1 h at 37°C, followed by incubation with a secondary HRP-conjugated goat anti-mouse IgG antibody (1:3,000 dilution) for 30 min at 37°C. Signal was detected with a TMB Liquid Substrate System. Samples were assayed in triplicate. A sample was considered as positive if the net OD value was above the set cut-off and at least 0.2 OD (Uusi-Kerttula et al., 2014).

### HBGAs Blockade Assay

Blockade assay was used to test the ability of antibodies blocking the binding of P particles to ligands. Briefly, microtiter plates were coated with PGM at 4°C overnight, followed by blocking with 5% skimmed milk. Two-fold diluted mouse sera (starting dilution 1:100) were pre-incubated with GII.17 P particle (1 μg/ml) for 1 h at 37°C. After that, adding the pre-incubated mixtures to the plates and then incubated for 1 h at 37°C. The plate were further incubated with immunized rabbit sera at the dilution of 1:2,000 for 1 h at 37°C, followed by a secondary HRP-conjugated goat anti-rabbit IgG antibody (1:3,000 dilution) at 37°C for 30 min. Signal was detected with the TMB Liquid Substrate System. Samples were assayed in triplicate. The blocking index (%) was calculated as 100% – [(OD wells with serum/OD wells without serum) × 100%; Tamminen et al., 2012]. Sera that did not block 50% of P particle binding to PGM at the lowest serum dilution tested were assigned a titer equal to 0.5 times the limit of detection for statistical analysis (Lindesmith et al., 2017).

### Statistical Analysis

All serological data are expressed as the geometric mean titers (GMT). Statistical analysis was performed with Graphpad prism8.0. Schapiro-Wilk test and Mann-Whitney U test were performed to assess the antibody binding properties. Significant differences from different groups were determined using One-way ANOVA. Value of *p* < 0.05 was statistically significant.

## Results

### Selection of Representative Strains of GII.17 Four Clusters

Phylogenetic relationships of the GII.17 strains based on their capsid protein sequences have been analyzed by our previous studies (Xue et al., 2016b,c). As a result, GII.17 NoV was divided into four clusters (GII.17-A, GII.17-B, GII.17-C, and GII.17-D) during the evolution process. Multiple amino acid sequence alignments showed that a total of 87 differences were found in the capsid protein sequences of four clusters, of which 21 sites were specific to the newly emerged GII.17 Kawasaki 2014 variant. In addition to amino acid changes, six deletion/insertion mutations were identified at site 296, 297, 344, 380, 386, and 400 (corresponding to the consensus capsid protein sequence of all GII.17 strains).

In this study, four representative strains of NoV GII.17 were selected as reference strains to explore the evolutionary mechanism: GII.17-A|2002|CS-E1 (GenBank accession number AY502009.1), GII.17-B|2005|Katrina-17 (GenBank accession number DQ438972.1), GII.17-C|2013|Saitama5203 (GenBank accession number LC043167.1), and GII.17-D|2015|GZ2015-L343 (GenBank accession number KT970376.1).

### Preparation of Capsid P Protein and Their Antisera Titer

**Figure 2:**
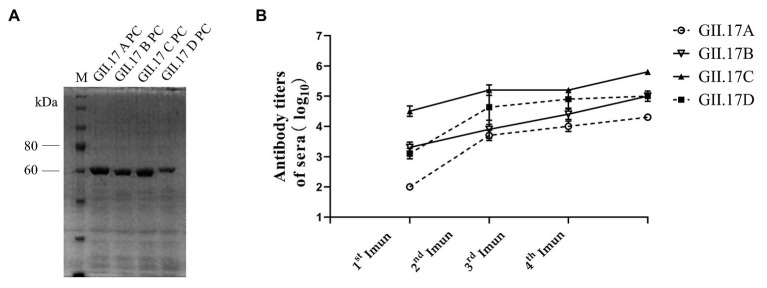
**(A)** SDS-PAGE analysis of capsid P protein of NoV GII.17 A, GII,17 B, GII.17C, and GII.17 D. The gel was stained with Brilliant Blue (G-250). The M lane contains protein standards. **(B)** Titers of NoV GII.17 sera antibodies. Sera samples were collected from mice immunized with GII.17 A, GII.17 B, GII.17 C, and GII.17 D fusion protein at weeks 2, 4, 5, and 6. Antibody titers were measured by indirect ELISA using a 1:200 dilution of sera samples, and the results are represented in log10. 1^st^ Imun, the first immunization; 2^nd^ Imun, the second immunization; 3^rd^ Imun, the third immunization; and 4^th^ Imun, the fourth immunization.

The P domain of representative strain clusters A (GII.172002), B (GII.172005), C (GII.172013), and D (GII.172014) were expressed for P particle production. As shown in Figure 2A, four GII.17 GST-fused capsid proteins eluted from the Glutathione Sepharose beads were 63 kDa. ELISA results showed that after each subsequent immunization, the titer of specific antibodies increased (Figure 2B). Immunization with GII.17 A, GII.17 B, GII.17 C, and GII.17 D GST-P fusion protein resulted in equally high titer of specific sera antibodies, with GMT values of 20, 000 (95% CI = 20, 000 – 20, 000); 100, 000 (95% CI = 37, 321 – 278, 576); 640, 000 (95% CI = 640, 000 – 640, 000); and 158, 489 (95% CI = 37, 321 – 278, 576), respectively.

### HBGA Binding Profile of Four GII.17 P Proteins

**Figure 3:**
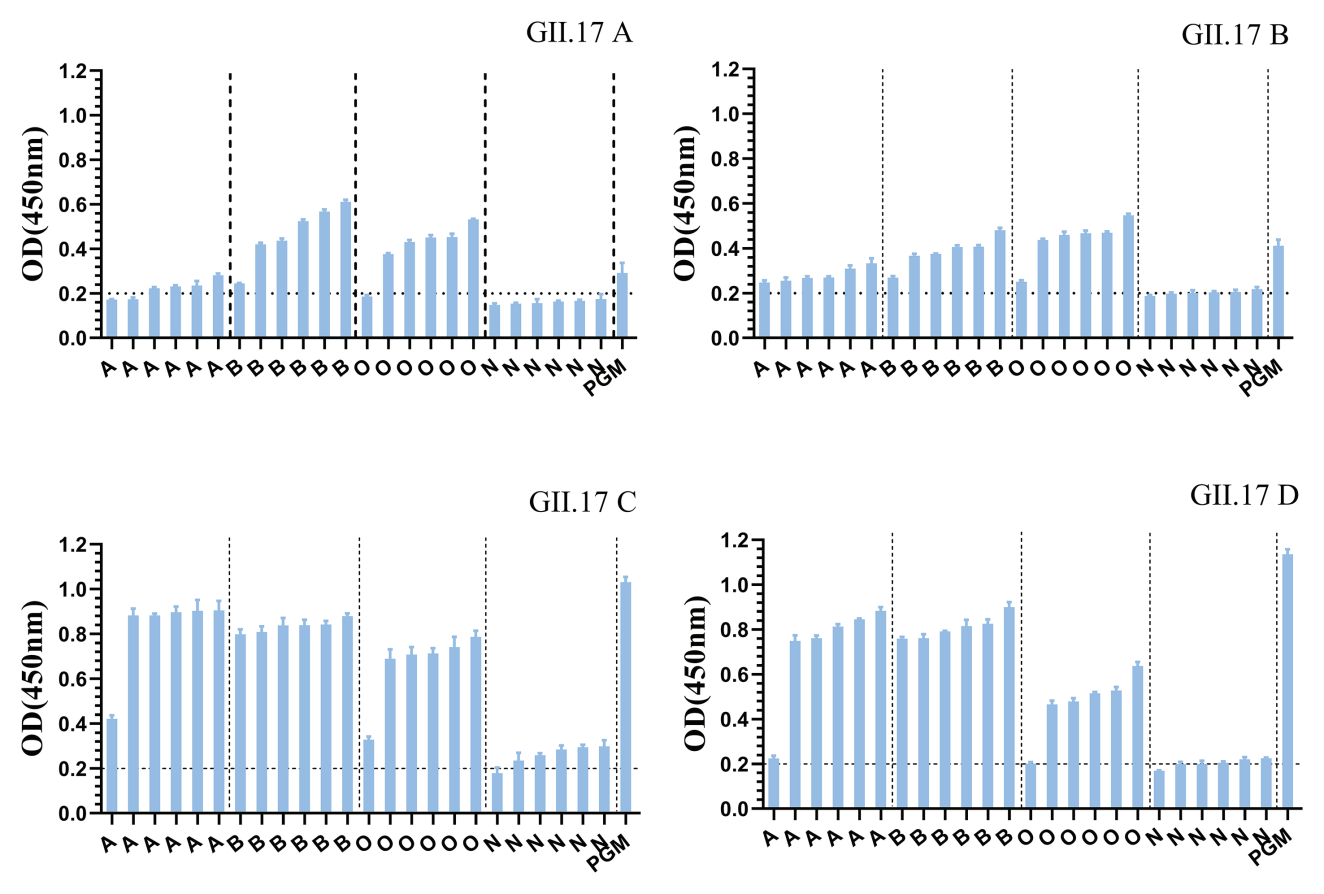
Binding of four clusters of GII.17 NoV P particles to saliva samples. GII.17 P particles were assayed for the binding property to saliva samples phenotyped by ELISA. Saliva samples from six individuals of each carbohydrate type (type A, B, O, and N), pig gastric mucin (PGM) were coated onto 96-well microplates, and then add P protein from four clusters of GII.17 viruses (GII.17 A, GII.17 B, GII.17 C, and GII.17 D). Shown are mean OD values with standard errors of P particles binding to saliva type A, B, O, non-secretor, and pig gastric mucin (PGM). Each group was sorted from the lowest to the highest in terms of the intensity of binding to saliva according to the OD 450 nm readings. Dotted line represents the cut-off value for samples considered positive. O, A, B, and N represent the type O (H antigen), A, B, and non-secretor saliva, respectively.

GII.17 P particles were assessed for their capacity to bind to cellular ligands, that is, HBGAs present in human saliva (types A, B, O, and non-secretor) and PGM (Figure 3). The results showed that limited binding or no binding signals were observed for the binding of previous strains (GII.17 A and GII.17 B) to HBGAs in the saliva samples of types A, B, and O, and to PGM. However, newly emerged GII.17 variants (GII.17 C and GII.17 D) showed significantly higher binding signals, and both previous and recent strains hardly binding to non-secretors.

### Cross-Reactivity of Four GII.17 Antisera

**Figure 4:**
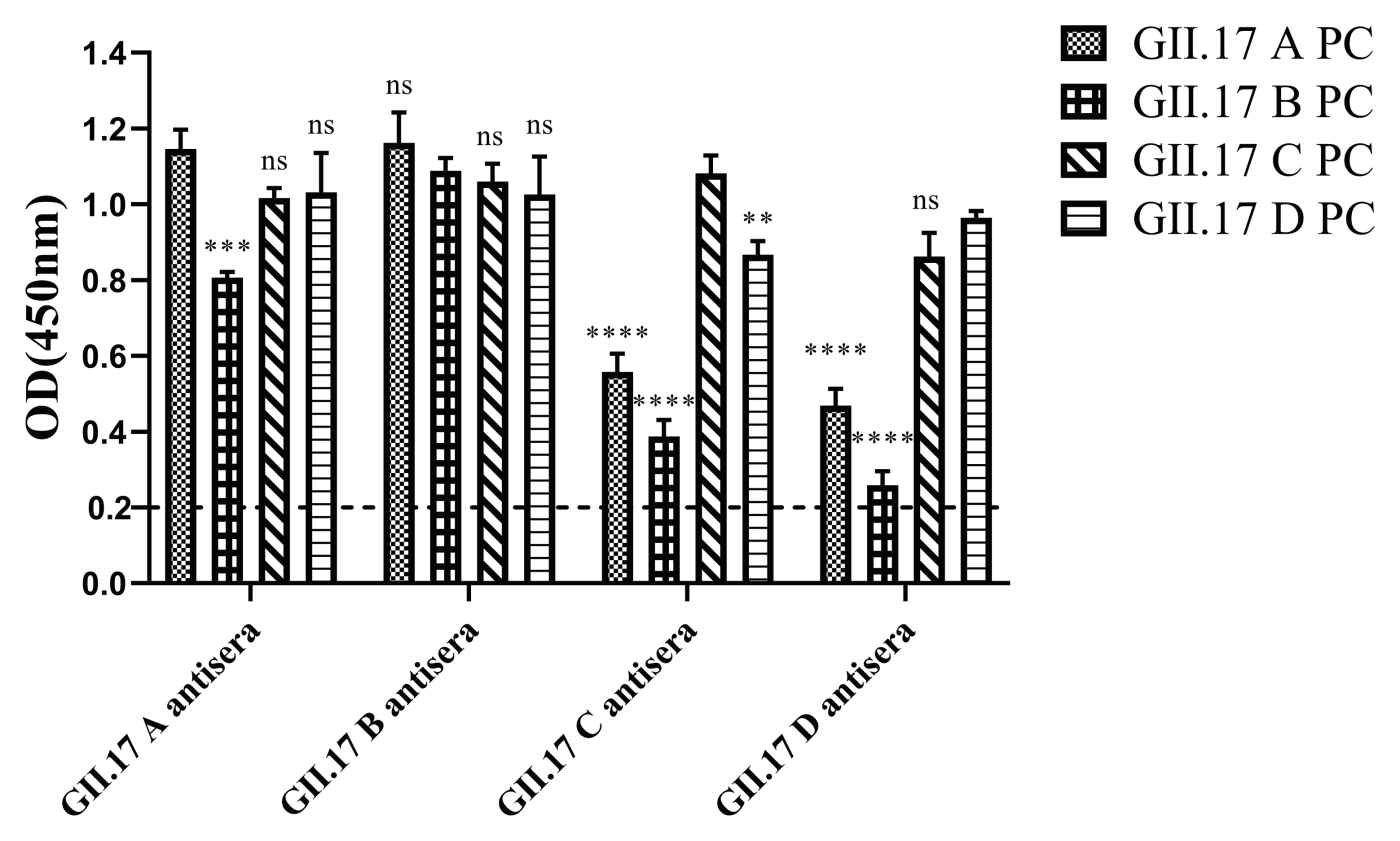
GII.17 antibody responses in homologous and heterologous mouse immune antisera. Antisera of GII.17 A, GII.17 B, GII.17 C, and GII.17 D were analyzed for cross-reactive responses. OD values were expressed as the mean ± SD. Dotted line represents the cut-off value for samples was considered as positive. ^*^Significantly different from homotypic sera (by ANOVA). ^*^p < 0.05; ^**^p < 0.01; ^***^p < 0.001; and ^∗∗∗∗^*p* < 0.0001. ns, not significant.

Immunized mouse sera were used to quantify type-specific and cross-reactive sera antibody responses. To determine the cross-reactivity of antibodies induced by the four clusters, sera were analyzed by indirect ELISA (Figure 4). Mice immunized with capsid P proteins from previous strains (GII.17 A and GII.17 B) induced relatively high cross-reactive responses against the other two cluster variants, with a minimum OD value of 0.8; however, the cross-reactivity of the antisera against the recent variants (GII.17-C and GII.17-D) with that against previous strains (GII.17-A and GII.17-B) showed a significant decrease (*p* < 0.0001), with a maximum OD value of 0.56. These results indicate that the major capsid proteins of the previous and recent GII.17 strains diverged from each other at the antigenic level.

### Preparation of GII.17 Mutants and Cross-Reactivity With Four Antisera

In addition to the changes in receptor binding function, spontaneous mutations in the capsid gene are also responsible for the evolution of GII.17 strains. This study compared nucleotide and amino acid sequences differences between the four representative GII.17 strains (Supplementary Table S1; Supplementary Figure S1). The identity of selected genomes at the nucleotide level was 85.4%. As a result of multiple amino acid sequence alignments, a total of 84 concrete differences in the capsid protein sequences of four strains were found. Based on the changes in amino acid sequences during the evolution of GII.17 NoV, we designed six mutants: 293-300A, 342-349A, 353-363A, 373-384A, 394-404A, and 444-454A.

**Figure 5:**
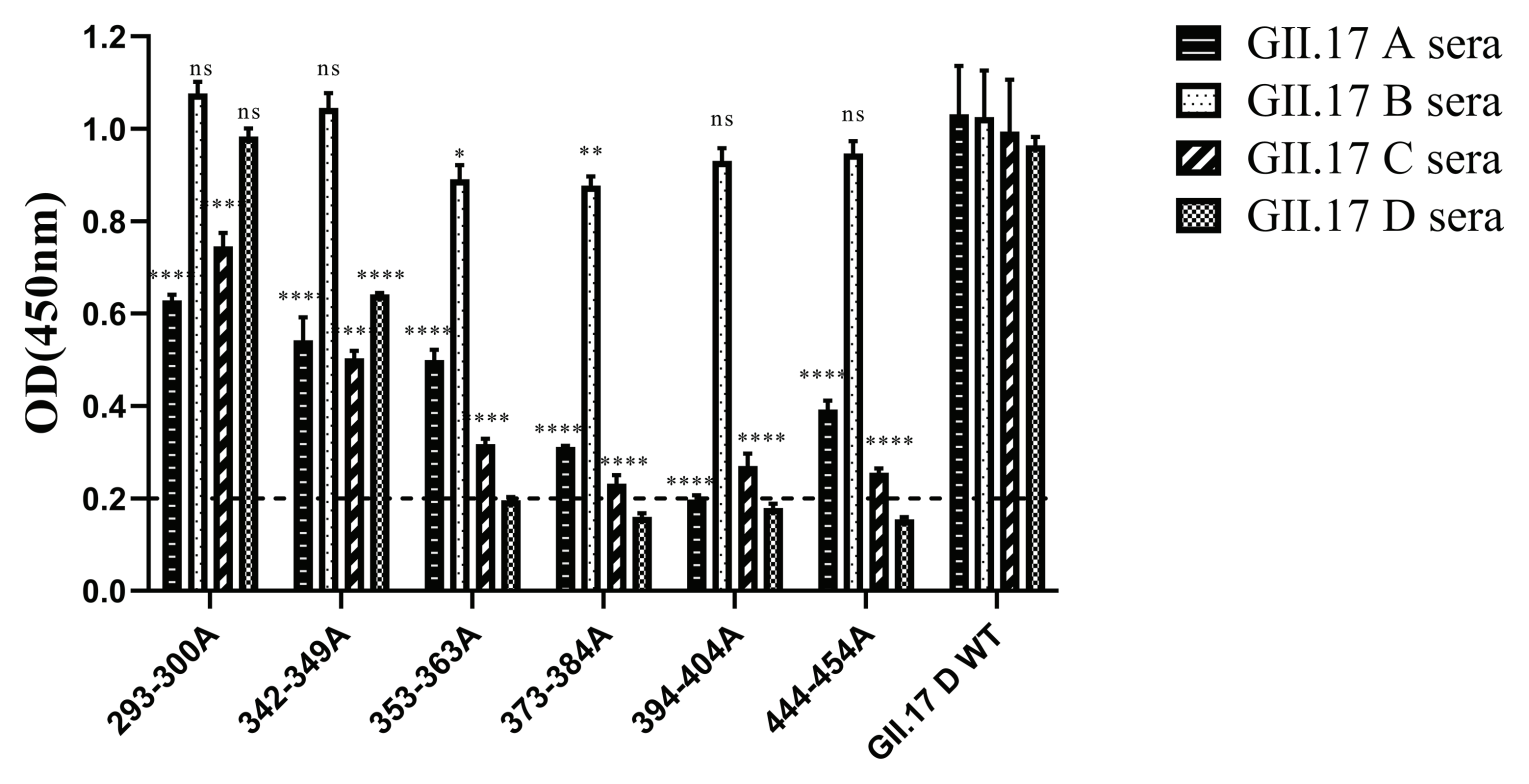
Cross-reactivity of six NoV GII.17 D mutants with GII.17 (A–D) antisera. OD values were expressed as the mean ± SD. Horizontal line illustrates the cut-off value for samples considered positive. ^*^Significantly different from wild-type GII.17 D variant sera (by analysis of variance). ^*^p < 0.05; ^**^p < 0.01; ^***^p < 0.001; and ^****^p < 0.0001. ns, not significant.

In our previous study about the variation of capsid proteins, the stability and binding ability of six GII.17 mutants (293-300A, 342-349A, 353-363A, 373-384A, 394-404A, and 444-454A) were verified by GII.17 monoclonal antibody. ELISA results showed that the six GII.17 mutants reacted with GII.17-2A111C1 monoclonal antibodies at OD value of 0.45 ± 0.07, 0.51 ± 0.08, 0.53 ± 0.02, 0.42 ± 0.06, 0.60 ± 0.17, and 0.48 ± 0.04, respectively, while the binding signal of positive control (GII.17 wild-type) and negative control were 0.46 ± 0.06 (*p* > 0.05) and 0.10 ± 0.04 (Unpublished data). The data indicated that mutation has no significant effect on the stability and binding ability of the P particles. ELISA results (Figure 5) showed that the cross-reactivity of all mutants tested with GII.17 A immunized sera decreased obviously, as is the case with GII.17 C immunized sera (*p* < 0.0001). Both 353-363A and 373-384A mutants had a robust decreased (*p* < 0.05 and *p* < 0.01, respectively) reaction with GII.17 B immunized sera, the other mutants have no obvious changes. As to GII.17 D immunized sera, the reaction of 293-300A mutant hardly changed; 353-363A, 373-384A, 394-404A, and 444-454A mutants had lost the binding property; 343-349A mutant had a significantly decreased reaction (*p* < 0.0001). In general, replacing residues 353-363aa, 373-384aa, 394-404aa, and 444-454aa had the greatest impact on cross-reactivity, followed by 293-300aa and 342-349aa.

### Cross-Clusters Blockade Antibody Responses of GII.17 Antisera

**Figure 6:**
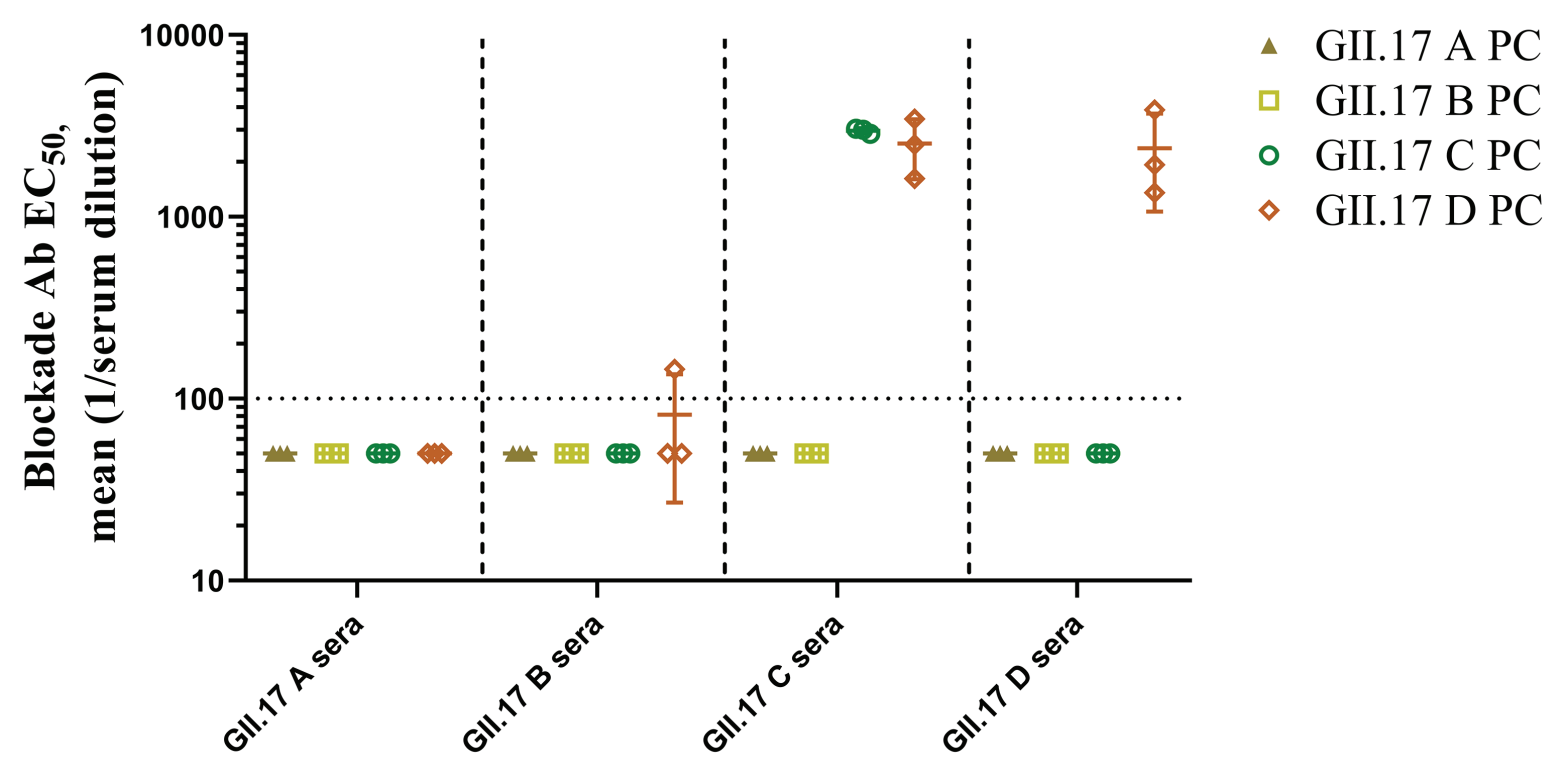
GII.17 type-specific and cross-reactive blockade antibodies. Sera from mice immunized with GII.17 P particles were evaluated for the ability to block P particle binding with carbohydrate ligand pig gastric mucin type III (PGM). The results are represented in log10 antibody titers. EC_50_, half-maximum effective concentration. Bars represent the geometric mean titer (GMT). Dotted line equals the assay limit of detection.

To measure the potential for cross-clusters protection as the viruses evolve, four GII.17 clusters and their antisera were tested for blockade activity. Since HGBAs binding ligands for GII.17 A and GII.17 B have not been identified, blockade potential for them could not be determined (Figure 6). As a result, GII.17 C and GII.17 D variants induced considerable homologous blocking activity as mean serum titers blocking at least 50% of P particle binding, with GMT of 3,162 (95% CI, 2,736–3,215) and 1,995 (95% CI, 160.3–3,942). GII.17 C antisera (GMT, 2,512; 95% CI, 253.2–4,793) were able to block GII.17 D P particle binding to PGM. Serum from mice immunized with GII.17 A and GII.17 B P particle was not able to block GII.17 C and GII.17 D variants. The result further suggested that major capsid protein of previous and recent GII.17 strains are undergoing antigenic variation over time.

## Discussion

NoV is the most common cause of acute viral gastroenteritis worldwide and GII.4 genotype variants have been predominant during the past two decades (Siebenga et al., 2009). However, GII.17 Kawasaki 2014 pandemic variants were found to cause an alarming number of outbreaks in certain parts of East Asian countries in 2014–2015 (Lu et al., 2015; Matsushima et al., 2015; Shi et al., 2016). The first GII.17 strain in the National Center for Biotechnology Information (NCBI) databank is from 1978 (Rackoff et al., 2013). Since then viruses with a GII.17 capsid genotype have sporadically been detected in Africa, Asia, Europe, North America, and South America (de Graaf et al., 2015).

When the novel GII.17 Kawasaki 2014 variant emerged and surpassed the predominating GII.4 viruses, there has been a concern of global spreading of GII.17 NoV (da Silva Ribeiro de Andrade et al., 2018). A previous study estimated that GII.17 VP1 evolves one order magnitude faster than GII.4 (Chan et al., 2015), which may allow their rapid emergence and cause a spread of a pandemic. Therefore, it is very important and necessary to evaluate the impact of sequence changes on antigenicity. This study further explored the mechanism behind the emergence and the high prevalence of the new variants.

The first GII.4 NoV emerged and caused a pandemic in the mid-1990s (Noel et al., 1999). Since 2002, new variants emerging every 2–4 years, resulting in epidemics and sometimes global pandemics (Siebenga et al., 2007; Huhti et al., 2014). A molecular evolution model has been proposed, that is, NoV GII.4 strains persist by evolving novel carbohydrate-binding domains in response to immune-driven selection and by antigenic drift in the receptor-binding regions of the P2 subdomain (Lindesmith et al., 2008). Monoclonal antibodies targeting distinct GII.4 strains show that antigenic variation is high and these strains are evolving in response to human herd immunity (Debbink et al., 2012b).

Multiple studies have shown that GII.4 NoV exhibited the broadest binding spectrum, attaching efficiently to saliva from secretor A, B, and O individuals, which may have contributed to the emergence of GII.4 viruses in human populations (de Rougemont et al., 2011; Dai et al., 2015). In this study, we characterized the binding spectrum of NoV GII.17 clusters to HBGAs in human saliva. Concordantly with the previous findings (Qian et al., 2019), recent GII.17 variants bound strongly to types A, B, O saliva and PGM, while previous GII.17 strains did not exhibit such strong binding. Strong binding of recent GII.17 variants to HBGAs in saliva samples of types A and B was observed, while binding to type O saliva were somewhat lower, especially GII.17 D (Malm et al., 2018). The HBGAs binding spectrum seemed to be consistent with the prevalence of the variants, in which the strains with strong binding capacity are more prevalent than those with weak binding, explaining the predominance of the new GII.17 variants. It further showed that the high prevalence of GII.17 D variant is the result of long-term evolution under the selection of HGBAs.

Antigenic change is another important factor that drives the evolution and the emergence of new variants that were often associated with new pandemics (Debbink et al., 2012a). Mice immunized with GST-P domain capsid proteins of the four clusters variants obtained high titer sera. Whether GST was removed from the immunogens or not did not significantly impact the immune responses (Wang et al., 2013). Previous strains antisera had high cross-reactivity against recent variants, with the minimum OD value of 0.8. However, sera from mice immunized with recent variants had a relatively weak reaction with previous strains, with the maximum OD value of 0.56. These results revealed that previous strains and recent variants were diverging from each other at the antigenic level, the significant antigenic epitopes of previous strains were still preserved and recent variants probably contain evolving antigenic epitopes and innovative structural changes to the capsid protein (Nilsson et al., 2003), such that changes probably lead to the evasion of the predominant protective immune response.

Receptor binding blockade assay with the results of sera from mice immunized with previous GII.17 strains had no blockade activity against recent GII.17 variants, combined with cross-reactivity data demonstrated that previous strains antisera may recognize highly conserved linear epitopes. Five blockade epitopes on VP1 in GII.17 NoV have been hypothesized, these are Epitope A (294–301aa, 374aa, and 378aa), Epitope B (336aa and 395aa), Epitope C (343aa and 382aa), Epitope D (residues 400–402aa), and Epitope E (residues 410aa, 414–416aa; Chen et al., 2015). Most variation we observed in the VP1 sequences of recent GII.17 strains mapped in or near these epitopes, which may lead to herd immune escape or alter human susceptibility to GII.17 NoV, thus partially explaining the prevalence of this novel variant (Chen et al., 2015).

To identify amino acids regulating antigenic differences over time, six GII.17 D mutants reacted with four GII.17 wild-type strains immunized serum. Mutational analysis showed that the mutations at amino acids 353–363, 373–384, 394–404, and 444–454 had the most significant impact on cross-reactivity, which further supported that those antigenic sites played an important role in promoting antigen evolution. In addition, we found that all wild-type variants and mutants had high cross-reactivity with GII.17 B antisera, and there is no significant difference in type-specific sera antibody response, which indicated that GII.17 B strain has strong potential for vaccine design.

This study explored the connection between antisera and different NoV GII.17 variants by immunizing mice. Although the mouse model is very extensive in immunological research, it will be more meaningful for using human antisera.

It is conceivable to anticipate cross-protection among GII.17 viruses since our data showed that GII.17 D variant immunized sera had a strong cross-reactivity response against the other clusters. Dai et al. (2017) found GII.4 antisera from patients had limited cross-reactivity or even no cross-blockade activity against GII.17, providing new evidence explaining the emergence and rapid expansion and indicating GII.17 variants need to be considered in future vaccine strategies. Important issues in NoV vaccine design are the genetic diversity and evolution resulting in escaping herd immunity, and our study provides a new perspective of them to produce broad protection against many GII.17 NoV variants. Understanding the molecular mechanism governing GII.17 antigenic variation is a key to design vaccines.

## Data Availability Statement

The datasets presented in this study can be found in online repositories. The names of the repository/repositories and accession number(s) can be found at: https://www.ncbi.nlm.nih.gov/genbank/, KT970376.1.

## Ethics Statement

The animal study was reviewed and approved by the Guangdong Institute of Microbiology Laboratory Animal Ethics Committee.

## Author Contributions

YZ and LX designed the experiments and wrote the manuscript. YJ and WC contributed reagents, materials, and analysis tools. JG, YLo, and YLn performed the experiments. ZQ and JY analyzed the data. MC and JW revised the manuscript. YD, JZ, and QW provided the project funding. All authors have read and agreed to the published version of the manuscript.

### Conflict of Interest

The authors declare that the research was conducted in the absence of any commercial or financial relationships that could be construed as a potential conflict of interest.
