# Supplementary material for: Evolutionary Mechanism of Immunological Cross-Reactivity Between Different GII.17 Variants
Source: Front Microbiol. 2021 Apr 6;12:653719. doi: 10.3389/fmicb.2021.653719 (PMC8055840; doi:10.3389/fmicb.2021.653719)
Supplement: Supplementary file 1 [file Image_1.pdf]

Supplementary Table 1 | Comparison of amino acid sequences between capsid proteins of four representative GII.17 strains.

site	16	22	24	40	75	169	171	173	174	234	244	258	281	289	290
GII.17-A|2002|CS-E1	T	I	N	P	I	N	Q	A	N	I	A	N	S	R	L
GII.17-B|2005|Katrina-17	A	I	T	A	V	N	R	S	E	I	A	N	T	K	I
GII.17-C|2013|Saitama5203	A	G	N	P	I	N	Q	N	S	L	V	Q	S	R	V
GII.17-D|2015|GZ2015-L343	A	G	N	P	I	S	Q	N	S	L	V	Q	S	R	V

site	291	**293**	**294**	**295**	**296**	**297**	**298**	**299**	**300**	301	307	313	314	318	330
GII.17-A|2002|CS-E1	T	D	V	D	G	S	H	D	D	R	T	P	F	E	T
GII.17-B|2005|Katrina-17	S	D	V	Q	N	S	H	Q	D	R	T	P	F	D	T
GII.17-C|2013|Saitama5203	T	E	T	D	-	-	H	R	D	K	Q	T	Y	D	K
GII.17-D|2015|GZ2015-L343	T	Q	I	N	-	-	Q	R	D	R	Q	T	Y	D	K

site	332	333	336	337	**342**	**343**	**344**	**345**	**346**	**347**	**348**	**349**	**353**	**354**
GII.17-A|2002|CS-E1	L	L	V	A	V	G	S	N	-	P	N	T	H	E
GII.17-B|2005|Katrina-17	L	L	V	A	V	G	G	T	G	N	N	T	H	E
GII.17-C|2013|Saitama5203	V	V	V	A	V	G	N	D	A	P	G	S	H	E
GII.17-D|2015|GZ2015-L343	V	V	M	V	V	G	N	D	A	P	G	S	Q	Q

site	**355**	**356**	**357**	**358**	**359**	**360**	**361**	**362**	**363**	371	**373**	**374**	**375**	**376**
GII.17-A|2002|CS-E1	A	V	V	S	T	T	S	S	Q	V	F	G	-	S
GII.17-B|2005|Katrina-17	V	V	I	A	T	T	S	T	Q	I	F	G	-	S

GII.17-C|2013| Saitama5203	A	V	I	S	T	Y	S	P	Q	V	F	R	-	S
GII.17-D|2015| GZ2015-L343	A	W	V	S	T	Y	S	P	Q	V	L	R	I	S
site	**377**	**378**	**379**	**380**	**381**	**382**	**383**	**384**	385	**394**	**395**	**396**	**397**	**398**
GII.17-A|2002| CS-E1	T	S	T	D	F	Q	L	Q	Q	I	K	-	I	E
GII.17-B|2005| Katrina-17	E	S	E	D	F	Q	V	G	P	I	K	-	I	E
GII.17-C|2013| Saitama5203	N	D	N	D	F	Q	L	-	Q	I	N	-	D	D
GII.17-D|2015| GZ2015-L343	D	N	D	D	F	Q	F	-	Q	V	S	D	D	D
site	**399**	**400**	**401**	**402**	**403**	**404**	407	408	410	414	425	440	**444**	**445**
GII.17-A|2002| CS-E1	S	G	H	E	F	D	A	L	R	H	I	N	A	G
GII.17-B|2005| Katrina-17	T	G	H	S	F	R	D	P	N	A	V	N	A	G
GII.17-C|2013| Saitama5203	G	D	H	P	F	R	E	L	D	L	V	F	S	G
GII.17-D|2015| GZ2015-L343	D	G	H	P	F	R	E	L	N	E	V	F	S	G
site	**446**	**447**	**448**	**449**	**450**	**451**	**452**	**453**	**454**	456	497	514	517	524
GII.17-A|2002| CS-E1	G	V	S	D	G	V	I	D	C	L	T	S	Y	V
GII.17-B|2005| Katrina-17	G	V	S	E	G	I	I	D	C	L	T	S	Y	I
GII.17-C|2013| Saitama5203	G	Y	N	Q	G	I	V	D	C	I	S	A	Y	V
GII.17-D|2015| GZ2015-L343	G	Y	N	Q	G	I	V	D	C	I	S	A	H	V

A dash represents the relative deletion of an amino acid at a certain position. Bold font style indicates the location of the mutation. The amino acid positions correspond to the sequence of GII.17-A|2002|CS-E1.

```
GII.17-A|2002|CS-E1           MKMASNDAAPSNDGATGLVPEINNETLPLEPVAGAAIAAPVTGQNNIIDPWIRTNFVQAPNGEFTVSPRNSPGEILLNLE     80
GII.17-B|2005|Katrina-17      MKMASNDAAPSNDGAAGLVPEINTETLPLEPVAGAALAAAVTGQSNIIDPWIRTNFVQAPNGEFTVSPRNSPGEVLLNLE     80
GII.17-C|2013|Saitama5203     MKMASNDAAPSNDGAAGLVPEGNNETLPLEPVAGAAIAAPVTGQNNIIDPWIRTNFVQAPNGEFTVSPRNSPGEILLNLE     80
GII.17-D|2015|GZ2015-L343     MKMASNDAAPSNDGAAGLVPEGNNETLPLEPVAGAAIAAPVTGQNNIIDPWIRTNFVQAPNGEFTVSPRNSPGEILLNLE     80
Consensus                     mkmasndaapsndga glvpe n etlplepvagaa aa vtgq niidpwirtnfvqapngeftvsprnspge llnle

GII.17-A|2002|CS-E1           LGPDLNPYLAHLSRMYNGYAGGVEVQVLLAGNAFTAGKILFAAVPPNFPVEFLSPAQITMLPHLIVDVRTLEPIMIPLPD    160
GII.17-B|2005|Katrina-17      LGPDLNPYLAHLSRMYNGYAGGVEVQVLLAGNAFTAGKILFAAVPPNFPVEFLSPAQITMLPHLIVDVRTLEPIMIPLPD    160
GII.17-C|2013|Saitama5203     LGPDLNPYLAHLSRMYNGYAGGVEVQVLLAGNAFTAGKILFAAVPPNFPVEFLSPAQITMLPHLIVDVRTLEPIMIPLPD    160
GII.17-D|2015|GZ2015-L343     LGPDLNPYLAHLSRMYNGYAGGVEVQVLLAGNAFTAGKILFAAVPPNFPVEFLSPAQITMLPHLIVDVRTLEPIMIPLPD    160
Consensus                     lgpdlnpylahlsrmyngyaggvevqvllagnaftagkilfaavppnfpveflspaqitmlphlivdvrtlepimiplpd

GII.17-A|2002|CS-E1           VRNTFFHYNNQPANRMRLVAMLYTPLRSNGSGDDVFTVSCRVLTRPTPDFEFTYLVPPSVESKTKPFSLPILTISELTNS    240
GII.17-B|2005|Katrina-17      VRNTFFHYNNRPSERMRLVAMLYTPLRSNGSGDDVFTVSCRVLTRPTPDFEFTYLVPPSVESKTKPFSLPILTISELTNS    240
GII.17-C|2013|Saitama5203     VRNTFFHYNNQPNSRMRLVAMLYTPLRSNGSGDDVFTVSCRVLTRPTPDFEFTYLVPPSVESKTKPFSLPILTLSELTNS    240
GII.17-D|2015|GZ2015-L343     VRNTFFHYSNQPNSRMRLVAMLYTPLRSNGSGDDVFTVSCRVLTRPTPDFEFTYLVPPSVESKTKPFSLPILTLSELTNS    240
Consensus                     vrntffhy n p  rmrlvamlytplrsngsgddvftvscrvltrptpdfeftylvppsvesktkpfslpilt seltns

GII.17-A|2002|CS-E1           RFPAPIDSLFTAQNNNLNVQCQNGRCTLDGELQGTTQLLPSGICAFRGRLTADVDGSHDDRWHMQLTNLNGTPFDPTEDV    320
GII.17-B|2005|Katrina-17      RFPAPIDSLFTAQNNNLNVQCQNGRCTLDGELQGTTQLLPTGICAFRGKISADVQNSHQDRWHMQLTNLNGTPFDPTDDV    320
GII.17-C|2013|Saitama5203     RFPVPIDSLFTAQNNVLQVQCQNGRCTLDGELQGTTQLLPSGICAFRGRVTAETDHRDKWHMQLQN..LNGTTYDPTDDV    318
GII.17-D|2015|GZ2015-L343     RFPVPIDSLFTAQNNVLQVQCQNGRCTLDGELQGTTQLLPSGICAFRGRVTAQINQRDRWHMQLQN..LNGTTYDPTDDV    318
Consensus                     rfp pidslftaqnn l vqcqngrctldgelqgttqllp gicafrg   a            q   lngt  dpt dv

GII.17-A|2002|CS-E1           PAPLGTPDFTGLLFGVASQRNVGSN.PNTTRAHEAVVSTTSSQFVPKLGSVNFGSTSTDFQLQQPTKFTPVGIKIESG.H    398
GII.17-B|2005|Katrina-17      PAPLGTPDFTGLLFGVASQRNVGGTGNNTTRAHEVVIATTSTQFVPKLGSINFGSESEDFQVGPPTKFTPVGIKIETG.H    399
GII.17-C|2013|Saitama5203     PAPLGTPDFKGVVFGVASQRNVGNDAPGSTRAHEAVISTYSPQFVPKLGSVNFRSNDNDFQLQP.TKFTPVGINDDGD.H    396
GII.17-D|2015|GZ2015-L343     PAPLGTPDFKGVVFGMVSQRNVGNDAPGSTRAQQAWVSTYSPQFVPKLGSVNLRISDNDDFQFQPTKFTPVGVNDDDDGH    398
Consensus                     paplgtpdf g  fg  sqrnvg      tra      t s qfvpklgs n      d      tkftpvg       h

GII.17-A|2002|CS-E1           EFDQWALPRYSGHLTLNMNLAPPIAPNFPGEQLLFFRSNVPCAGGVSDGVIDCLLPQEWIQHFYQESAPSQSDVALIRYV    478
GII.17-B|2005|Katrina-17      SFRQWDPPNYSGALTLNMNLAPPVAPNFPGEQLLFFRSNVPCAGGVSEGIIDCLLPQEWIQHFYQESAPSQSDVALIRYV    479
GII.17-C|2013|Saitama5203     PFRQWELPDYSGLLTLNMNLAPPVAPNFPGEQLLFFRSFVPCSGGYNQGIVDCLIPQEWIQHFYQESAPSQSDVALIRYV    476
GII.17-D|2015|GZ2015-L343     PFRQWELPNYSGELTLNMNLAPPVAPNFPGEQLLFFRSFVPCSGGYNQGIVDCLIPQEWIQHFYQESAPSQSDVALIRYV    478
Consensus                      f qw  p ysg ltlnmnlapp apnfpgeqllffrs vpc gg   g  dcl pqewiqhfyqesapsqsdvaliryv

GII.17-A|2002|CS-E1           NPDTGRTLFEAKLHRTGYITVAHSGDYPLVVPSNGYFRFDSWVNQFYSLAPMGTGNGRRRV                        539
GII.17-B|2005|Katrina-17      NPDTGRTLFEAKLHRTGYITVAHSGDYPLVVPSNGYFRFDSWINQFYSLAPMGTGNGRRRV                        540
GII.17-C|2013|Saitama5203     NPDTGRTLFEAKLHRSGYITVAHSGDYPLVVPANGYFRFDSWVNQFYSLAPMGTGNGRRRA                        537
GII.17-D|2015|GZ2015-L343     NPDTGRTLFEAKLHRSGYITVAHSGDYPLVVPANGHFRFDSWVNQFYSLAPMGTGNGRRRA                        539
Consensus                     npdtgrtlfeaklhr gyitvahsgdyplvvp ng frfdsw nqfyslapmgtgngrrr
```

**Supplementary Figure 1 | ** Comparison of nucleotide sequences of four GII.17 reference strains. A dot represents the relative deletion of a nucleotide at a certain position. 75% sequence identity among sequences are marked in red and 50% sequence identity are marked in yellow.
